# 1399. Clarithromycin–Rifampin-based Treatment for Non-tuberculous Mycobacterial Infections in Immunocompromised Patients Who Require Concomitant CYP-Metabolized Medications

**DOI:** 10.1093/ofid/ofab466.1591

**Published:** 2021-12-04

**Authors:** Isabel H Gonzalez-Bocco, Muneerah M Aleissa, Matthew Cheng, Jennifer Manne-Goehler, Francisco M Marty

**Affiliations:** 1 Brigham and Women’s Hospital, Chestnut Hill, Massachusetts; 2 McGill University Health Centre, Montreal, Quebec, Canada; 3 Massachusetts General Hospital, Dana-Farber Cancer Institute, Harvard Medical School, Boston, Massachusetts

## Abstract

**Background:**

Non-tuberculous mycobacteria (NTM) are causes of pulmonary and extrapulmonary disease that frequently affect immunocompromised hosts (ICH). Current treatment guidelines recommend a macrolide-based, multi-drug regimen that includes rifampin. Rifampin is a potent cytochrome P450 (CYP) 3A inducer, which often results in drug-drug interactions in ICH receiving multiple CYP substrates. One way to mitigate rifampin’s CYP induction is to utilize clarithromycin, a CYP inhibitor, as the accompanying macrolide. We evaluated the incidence of NTM treatment-related adverse events (AEs) in patients who received a clarithromycin-based regimen compared to patients who received an azithromycin-based regimen.

**Methods:**

We conducted a retrospective review of NTM infection in 30 immunocompromised adults. All participants had a positive culture for a NTM and had received a rifamycin (rifampin or rifabutin) with a macrolide (azithromycin or clarithromycin) for treatment at Brigham and Women’s Hospital between 01/01/2011-10/18/2020 or Dana-Farber Cancer Institute between 06/03/2015-07/01/2020. The primary outcome was the incidence of NTM treatment-related AEs in patients who received a clarithromycin-based regimen compared to those who received an azithromycin-based regimen.

**Results:**

There were no significant differences in the reasons for discontinuation of NTM treatment or 90-day mortality between groups. The number of AEs possibly related to NTM treatment were similar in patients who received a clarithromycin-based regimen and those who received an azithromycin-based one (10/13 vs. 14/17; p=0.73). The most common AE was liver function test abnormalities (Table 1). Additionally, the proportion of patients requiring dose adjustments for interacting medications and patients with out-of-range tacrolimus levels were similar between the two groups (23.1% vs. 29.4%; p=0.76 and 8.0% vs. 6.0%; p=1.00, respectively).

Table 1: Adverse events

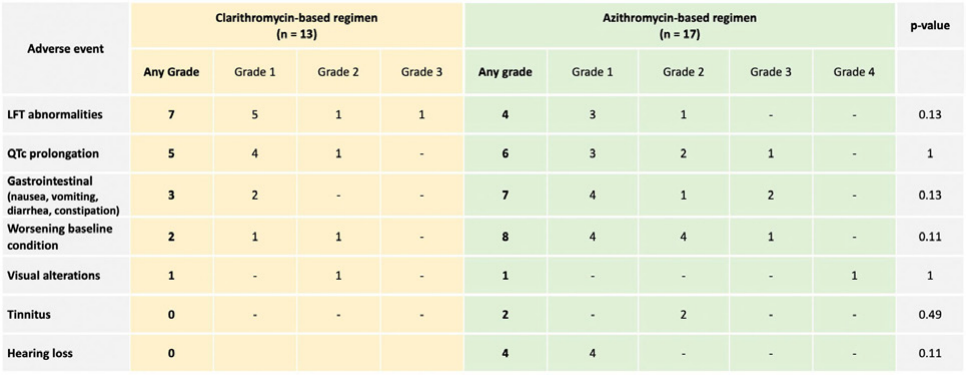

**Conclusion:**

A clarithromycin-based regimen for NTM treatment was safe and well tolerated in our patient population. This combination provides a good alternative for patients requiring medications that are CYP substrates, or those who cannot tolerate azithromycin.

**Disclosures:**

**Matthew Cheng, MD**, **GEn1E Lifesciences** (Advisor or Review Panel member)**Kanvas Biosciences** (Board Member, Shareholder)**nplex biosciences** (Advisor or Review Panel member)

